# Draft Genome Sequence of *Chromobacterium violaceum* Strain CV017

**DOI:** 10.1128/genomeA.00080-16

**Published:** 2016-03-03

**Authors:** Xiaofei Wang, Kara C. Hinshaw, Stuart J. Macdonald, Josephine R. Chandler

**Affiliations:** a Department of Molecular Biosciences, University of Kansas, Lawrence, Kansas, USA; b Center for Computational Biology, University of Kansas, Lawrence, Kansas, USA

## Abstract

We announce the draft genome sequence for *Chromobacterium violaceum* strain CV017, used as a model and tool to understand acyl-homoserine lactone-dependent quorum sensing. The assembly consists of 4,774,638-bp contained in 211 scaffolds.

The soil saprophyte *Chromobacterium violaceum* is an occasional human pathogen known for its ability to produce a biotechnologically interesting toxic purple pigment, violacein (1, 2). Strain CV017, derived from a soil isolate (ATCC 31532) (3), has a transposon insertion at an unknown site causing overproduction of violacein (4). Violacein is controlled by acyl-homoserine lactone-dependent quorum sensing, and derivatives of CV017 have been widely utilized as acyl-homoserine lactone biosensors (5). Because of its growth characteristics and production of quorum-dependent antimicrobials, CV017 is also useful to understand the role of quorum sensing in interspecies competition (6). To date, only one other *C. violaceum* strain has been sequenced, ATCC 12472 (accession NC_005085.1) (7). Here we report the draft genome sequence of the genetically distinct strain CV017.

CV017 cells were grown in Luria-Bertani media, and genomic DNA was isolated using the Gentra Puregene Bacteria/Yeast kit (Qiagen). The DNA was used to make a sequencing library with 1-kb inserts, and was sequenced on an Illumina MiSeq generating 300-bp paired-end reads. Raw reads were preprocessed with Scythe v0.991(https://github.com/vsbuffalo/scythe) and Sickle v1.200 (https://github.com/najoshi/sickle) to improve read quality, aligned to the phiX174 genome via Bowtie2 v2.1.0 (8) to remove any contaminating reads, and assembled with ABySS v1.9.0 (9) using an empirically determined optimal k-mer size of 115. The resulting assembly consists of 211 scaffolds, has an *N*_50_ scaffold size of 40,489-bp, a summed length of 4,774,638-bp, and read coverage of the scaffolds is around 1,250×. G+C content of the sequence is 64.5%.

We annotated the assembly using NCBI Prokaryotic Genomes Annotation Pipeline (PGAP) (http://www.ncbi.nlm.nih.gov/genome/annotation_prok/), identifying 4,178 potential CDS (coding DNA sequences) on 168/211 scaffolds, and assigning a putative function to 2,559 (61%) CDS—the remainder are classified as hypothetical proteins. We additionally identified 1 noncoding RNA (ncRNA), 105 tRNA, 46 complete rRNA genes, and 3 clustered regularly interspaced short palindromic repeat (CRISPR) arrays.

Comparison of the CV017 scaffolds to the genome strain ATCC 12472 using MUMmer (10) revealed that 64.4% of the bases in ATCC 12472 were covered by assembled scaffolds. Outside of these regions, nucleotide conservation between the two strains appears to be modest. We searched the CV017 draft genome to identify the putative transposon, previously described as a mercury-resistant mini-Tn5 (4). We found four mercury resistance genes (*merBAPT*) flanked by the conserved 19-bp Tn5 terminal ends. This putative transposon element is located 66-bp upstream of the predicted ATG start site of a CDS located between 11,559 and 11,975-bp on scaffold 184, a homolog of CV_1055 encoding a protein of unknown function in ATCC 12472. The sequence resembles the mini-Tn5 Hg^r^ from plasmid pUT/Hg (11). Interestingly, in another study nine transposon mutants of the parental strain ATCC 31532 that had a similar phenotype as CV017 (enhanced purple pigmentation) also mapped to a homolog of CV_1055 (12). Likely, the mini-Tn5 in CV017 is inserted into the promoter region and disrupts expression of the CV_1055 homolog. Future efforts by our group will focus on using this draft genome assembly to find quorum sensing-controlled genes that are important for interspecies competition in CV017.

## Nucleotide sequence accession numbers.

This whole-genome shotgun project has been deposited at DDBJ/EMBL/GenBank under the accession number LKIW00000000. The version described in this paper is version LKIW01000000.
